# HPV in women assisted by the Family Health Strategy

**DOI:** 10.11606/S1518-8787.2017051000065

**Published:** 2017-11-07

**Authors:** Andréia Rodrigues Gonçalves Ayres, Gulnar Azevedo e Silva, Maria Teresa Bustamante Teixeira, Kristiane de Castro Dias Duque, Maria Lúcia Salim Miranda Machado, Carmen Justina Gamarra, José Eduardo Levi

**Affiliations:** I Hospital Universitário Gaffrée e Guinle. Universidade Federal do Estado do Rio de Janeiro. Rio de Janeiro, RJ, Brasil; II Departamento de Epidemiologia. Instituto de Medicina Social. Universidade do Estado do Rio de Janeiro. Rio de Janeiro, RJ, Brasil; III Núcleo de Assessoria, Treinamento e Estudos em Saúde. Universidade Federal de Juiz de Fora. Juiz de Fora, MG, Brasil; IV Faculdade de Saúde Coletiva. Universidade Federal da Integração Latino-Americana. Foz do Iguaçu, PR, Brasil; V Laboratório de Virologia. Instituto de Medicina Tropical. Universidade de São Paulo. São Paulo, SP, Brasil

**Keywords:** Papillomaviridae, Prevalence, Mass Screening, Uterine Cervical Neoplasms, prevention & control, Family Health Strategy, Papillomaviridae, Prevalência, Programas de Rastreamento, Neoplasias do Colo do Útero, prevenção & controle, Estratégia Saúde da Família

## Abstract

**OBJECTIVE:**

Estimate the prevalence of cervical HPV infection among women assisted by the Family Health Strategy and identify the factors related to the infection.

**METHODS:**

A cross-sectional study involving 2,076 women aged 20–59 years old residing in Juiz de Fora, State of Minas Gerais, who were asked to participate in an organized screening carried out in units were the Family Health Strategy had been implemented. Participants answered the standardized questionnaire and underwent a conventional cervical cytology test and HPV test for high oncogenic risk. Estimates of HPV infection prevalence were calculated according to selected characteristics referenced in the literature and related to socioeconomic status, reproductive health and lifestyle.

**RESULTS:**

The overall prevalence of HPV infection was 12.6% (95%CI 11.16–14.05). The prevalence for the pooled primer contained 12 oncogenic HPV types (31, 33, 35, 39, 45, 51, 52, 56, 58, 59, 66, and 68) was 8.6% (95%CI 7.3–9.77). In the multivariate analysis, it was observed that the following variables were significantly associated with a higher prevalence of HPV infection: marital status (single: adjusted PR = 1.40, 95%CI 1.07–1.8), alcohol consumption (any lifetime frequency: adjusted PR = 1.44, 95%CI 1.11–1.86) and number of lifetime sexual partners (≥ 3: adjusted PR = 1.35, 95%CI 1.04–1.74).

**CONCLUSIONS:**

The prevalence of HPV infection in the study population ranges from average to particularly high among young women. The prevalence of HPV16 and HPV18 infection is similar to the worldwide prevalence. Homogeneous distribution among the pooled primer types would precede the isolated infection by HPV18 in magnitude, which may be a difference greater than the one observed. The identification of high-risk oncogenic HPV prevalence may help identify women at higher risk of developing preneoplastic lesions.

## RESUMO

**OBJETIVO:**

Estimar a prevalência de infecção do colo do útero pelo HPV entre mulheres assistidas pela Estratégia Saúde da Família e identificar os fatores relacionados à infecção.

**MÉTODOS:**

Trata-se de estudo transversal, no qual participaram 2.076 mulheres de 20 a 59 anos, residentes em Juiz de Fora, MG, convocadas para rastreamento organizado, realizado em unidades com a Estratégia Saúde da Família implantada. As participantes responderam ao questionário padronizado, realizando exame citológico cervical convencional e teste para HPV de alto risco oncogênico. Foram calculadas estimativas de prevalência de infecção pelo HPV segundo características selecionadas, referenciadas na literatura, relacionadas ao status socioeconômico, saúde reprodutiva e estilo de vida.

**RESULTADOS:**

A prevalência global de infecção pelo HPV foi 12,6% (IC95% 11,16–14,05). A prevalência para o *pooled primer* contendo 12 tipos de HPV oncogênicos (31, 33, 35, 39, 45, 51, 52, 56, 58, 59, 66 e 68) foi 8,6% (IC95% 7,3–9,77). Na análise multivariada, observou-se que as seguintes variáveis estavam significativamente associadas a uma maior prevalência de infecção por HPV: estado conjugal (solteira: RP ajustada = 1,40; IC95% 1,07–1,8), consumo de bebidas alcoólicas (qualquer frequência durante a vida: RP ajustada = 1,44; IC95% 1,11–1,86) e número de parceiros sexuais ao longo da vida (≥ 3: RP ajustada = 1,35; IC95% 1,04–1,74).

**CONCLUSÕES:**

A prevalência de infecção pelo HPV na população estudada varia de média a particularmente alta entre as mulheres jovens. A prevalência de infecção por HPV16 e HPV18 se assemelha às mundiais. Uma distribuição homogênea entre os tipos do *pooled primer* precederia a infecção isolada pelo HPV18 em magnitude, podendo ser a diferença maior que a observada. A identificação da prevalência de HPV de alto risco oncogênico pode auxiliar na identificação de mulheres sob maior risco de evolução para lesão preneoplásica.

## INTRODUCTION

Human papillomavirus (HPV) infection is one of the most common genital infections in the world and is a necessary cause for cervical cancer^10^. In Brazil, it is estimated that 15,590 women develop the disease each year, with a gross incidence rate of 15.33/100,000, which makes prevention and control of cervical cancer a priority in the health management pacts aimed at women’s health^9^.

The global strategy is the screening of pre-invasive lesions with a cervical cytological examination by smear exam. In developing countries, the impact of introducing and scaling up the screening as a health policy was lower than in developed countries, due to poor organization, poor coverage, and lack of quality assurance^21^. In the fastest-developing countries of South America, there was a decline in cervical cancer mortality, with estimates of the annual percentage change ranging from -1.4 to -6.3 between 1983 and 2002^29^. In Latin America, this tendency occurs in countries with higher average incomes, such as Argentina and Uruguay, and in countries with a history of implementing more organized screening, such as Mexico, Colombia, Chile, and Costa Rica, where mortality rates were reduced almost in half, causing an inversion in the in situ/invasive cancer ratio^19^. In Brazil, there has been an overall decline in cervical cancer mortality rates in the last few decades, but heterogeneously in the different macro-regions (annual percentage changes range from -3.3 to 1.7), showing partial success^12^. The limitations of screening include poorer access mainly among low-income women, the difficulty of following-up women’s examination in a centralized registration system, and the recruitment of women is performed in an unorganized way that does not follow the periodicity recommendation^22^.

These failures motivated the search for alternative techniques (associated or substitute) that would contribute to the reduction of losses and more accurate identification of women with HPV types with higher oncogenic risk. Molecular biology techniques have contributed to understanding genital HPV infection in different settings, and are used in scientific research since the beginning of the 1980s, but only recently have been incorporated into the health services^5^ ^,^ ^30^ ^,^ ^31^.

In Brazil, the identification of HPV-infected women is based on heterogeneous studies and, therefore, is of difficult comparability and limited reproducibility since estimates made from studies that consider referenced women may result in overestimated measures^2^. Prevalence estimates and factors associated with HPV infection make it possible to understand why certain groups of women are more vulnerable than others in order to make proposals for specific prevention actions for such groups. These proposals seek to increase the effectiveness of primary and secondary cervical cancer prevention. This type of cancer is an avoidable disease^14^.

Thus, the objective of the present study was to estimate the prevalence and risk factors for HPV infection among women residing in the coverage area of the Family Health Strategy (FHS).

## METHODS

A cross-sectional study was carried out with a population of residents of an area attached to the two units of the Family Health Strategy located on the outskirts of the city of Juiz de Fora, state of Minas Gerais. All women residing in the FHS coverage area between 20 and 59 years old, asymptomatic, were considered eligible. Pregnant women, those who were immunocompromised or had been previously submitted to uterus excision procedures (hysterectomy, conization) were excluded. Approximately 3,500 women were recruited in their homes by community health agents to undergo the preventive examination in the proposed area as a programmatic action developed by the family health units, with an emphasis on the organized screening of absences through the FHS register.

Data were collected from September 2010 to August 2012. Study participants were interviewed by trained health professionals, using a standardized questionnaire adapted from the National Health Survey, applied in the Federal District in 2010 and improved from 2013 onwards through the partnership between the Oswaldo Cruz Foundation and the Brazilian Institute of Geography and Statistic. The final instrument encompassed question groups distributed in nine modules: identification, sociodemographic characteristics, social support, self-assessment of health status, lifestyle, morbidity, women’s health, sexual behavior, and sexually transmitted infections. These data were measured. The instrument, its detailing, and the definition of each category used are available for consultation. Anthropometric data and morbidity data (blood pressure) were measured, and the results of exams refer to reports presented by participants^6^.

The women were then submitted to conventional cervical cytology examination at the primary health units, while simultaneously being tested for HPV infection, and the samples were collected by the nurses and trained physicians working in the units.

HPV testing was performed at the Laboratory of Virology of the Institute of Tropical Medicine of the Universidade de São Paulo. The uterine cervix samples were preserved in a PreservCyt solution and analyzed by the Polymerase Chain Reaction (PCR) method using the HPV-HR test + GT 16/18 test on the cobas 4800 HPV Test^®^ automated platform (Roche Molecular Systems, Inc., Branchburg, New Jersey, USA), which uses primers to amplify the DNA of 14 high-risk HPV types: HPV16, HPV18 and a pooled primer with 31, 33, 35, 39, 45, 51, 52, 56, 58, 59, 66, e 68 HR-HPV. Negative results were those specimens in which there was no amplification of viral DNA and positive ones were those in which there was an amplification of the viral DNA corresponding to the test primers. The procedures for analyzing the sample quality occurred according to pre-established protocols adopted by the executing Laboratory.

In the study period, 2,076 eligible women, approximately 60% of the population of women residing in the areas assigned to the FHS, answered to the recruitment, attended the FHS units and were included in the study. Due to reading problems, it was not possible to obtain HPV test results for 54 women (2.6%), because 40 samples had invalid results and 14 had inconclusive results. Thus, the study population had 2,022 women. In one of the health units, the number of women studied was almost twice the one obtained in the other unit, maintaining the proportionality of the size of the populations assigned to these two units.

We performed a univariate analysis of the data, and the absolute and relative frequencies were calculated for the nominal categorical variables, according to the family health unit. Then, the bivariate analysis was performed parallel to the comparison of the basic characteristics between the groups, using the chi-square test for proportions, which were considered significant if p ≤ 0.20. We measured the HPV infection prevalence, general and stratified according to selected variables with respective 95% confidence intervals (95%CI) and p value.

To evaluate the factors related to the infection, identified as the positive result for the HPV test, variables were selected according to epidemiological criteria and biological plausibility. Included in the study were characteristics related to: age, schooling, skin color, *per capita* family income, health evaluation, lifestyle (alcohol consumption, current and previous smoking), reproductive history (age at menarche, use of contraceptive methods, preventive screening, parity) and sexual behavior (age at sexarche, number of sexual partners throughout life, history of sexually transmitted infections). The variable categories were defined according to classic literature references, selected in the development of the questionnaire. The variable *per capita* income was categorized based on the minimum wage values at the time of the study (R$603.31, which was approximated to R$600.00). This way, low income corresponded to values below 50% of the minimum wage, average income corresponded to values between 50% and 100% minimum wage and high income was more than one minimum wage. The total income of the family, including all residents of the household, was considered.

Gross and adjusted prevalence ratios were calculated using Poisson regression with robust variance and respective 95% confidence intervals. The variables that in the bivariate analysis had p ≤ 0.20 were selected for the final model, which, in addition, included the age group and health unit of the family in which the woman resided and was assisted. All analyses were performed using the statistical software Stata, version 12 (Data Analysis and Statistic Software, StataCorp LP, College Station, Texas, USA).

The project was approved by the Ethics Committee of the Institute of Social Medicine of the Universidade do Estado do Rio de Janeiro (Position 0026.1.259.180-09), following all the proposed recommendations. All the women included in the study did so by reading and signing the informed consent form, ensuring the confidentiality of the data and the privacy of the women participating in all the stages of the study.

## RESULTS

The sociodemographic characteristics, as well as those related to the self-assessment of health, lifestyle, reproductive health and sexual behavior of the women studied, are described in Table 1.

**Table 1:** Sociodemographic characteristics, housing conditions, health self-assessment, lifestyle, reproductive health and sexual behavior among women living in the coverage area of the Family Health Strategy. Juiz de Fora, State of Minas Gerais, 2010–2012.

Variable	Category	Unit I		Unit II		Total	
		
		n	%	n	%	n^a^	%
Age group (years)	20–24	151	11.6	107	13.9	258	12.5
	25–29	181	14.0	98	12.8	279	13.5
	30–34	179	13.8	124	16.2	303	14.7
	35–39	175	13.5	105	13.7	280	13.6
	40–44	167	12.9	107	13.9	274	13.3
	45–49	157	12.1	89	11.6	246	11.9
	50–54	149	11.5	73	9.5	222	10.8
	55–59	136	10.5	64	8.3	200	9.7
Marital status	Single	296	22.9	132	17.2	428	20.8
	Had a partner	998	77.1	635	82.8	1,633	79.2
Years of study	< 1	8	0.6	13	1.7	21	1.0
	1–3	89	6.9	50	6.6	139	6.8
	4–7	474	36.9	298	39.4	772	37.8
	8–10	257	20.0	167	22.1	424	20.8
	> 11	456	35.5	228	30.1	684	33.5
Skin color	White	628	48.5	342	44.6	970	47.0
	Non-white	667	51.5	425	55.4	1,092	53.0
Religious practice	Yes	1,260	99.1	728	96.8	1,988	98.3
	No	11	0.9	24	3.2	35	1.7
*Per capita* income	Low	478	36.9	299	39.0	777	37.7
	Medium	771	59.5	443	57.8	1,214	28.9
	High	46	3.5	25	3.3	71	7.4
Piped water	General network	1,283	99.5	748	97.5	2,031	98.7
	Other	7	0.5	19	2.5	26	1.3
Waste destination	Regular waste collection	1,286	99.5	753	98.2	2,039	99.0
	Others	7	0.54	14	1.8	21	1.0
Outlet	Sewerage system	1,266	97.8	700	91.3	1,966	95.3
	Others	29	2.2	67	8.7	96	4.7
Health assessment	Poor to regular	525	40.7	336	43.8	861	41.9
	Good to very good	764	59.3	431	56.2	1,195	58.1
Alcohol consumption	No	698	53.9	481	62.7	1,179	57.2
	Yes	597	46.1	286	37.3	883	42.8
Current smoker	No	986	77.2	616	80.4	1,602	78.4
	Yes	291	22.8	150	19.6	441	21.6
Former smoker	No	728	71.0	459	72.6	1,187	71.6
	Yes	298	29.0	173	27.4	471	28.7
Age at menarche	≤ 12 years	589	46.2	316	42.1	905	44.6
	≤ 13 years	687	53.8	435	57.9	1,122	55.3
Examination	3 years ago, or less	1,016	78.5	585	76.3	1,601	77.7
Pap smear	Delayed^b^/Never had	278	21.5	182	23.7	460	22.3
Contraception	Any method	872	67.6	536	69.9	1,408	68.4
	No method	418	32.4	231	30.1	649	31.4
Nulliparity	No	1,134	87.6	669	87.3	1,803	87.5
	Yes	161	12.4	97	12.7	258	12.5
Age at sexarche experience	≥ 16 years	278	21.6	170	22.7	448	22.0
	≤ 15 years	1,008	78.4	580	77.3	1,588	78.0
Sexual partners^c^	Up to 3	903	73.1	530	72.3	1,433	72.8
	Over 3	333	26.9	203	27.7	536	27.2
Syphilis testing	Negative	635	98.3	315	98.4	950	98.3
	Positive	11	1.7	5	1.6	16	1.7
HIV testing	Negative	825	99.6	456	98.9	1,281	99.4
	Positive	3	0.4	5	1.1	8	0.6

^a^ Total of women with HPV test results and valid information about the variable.

^b^ Cervical cytology test performed more than three years ago.

^c^ Throughout life.

The overall prevalence of infection by at least one high-risk type of HPV among women of both FHS units was 12.6% (95%CI 11.16–14.05). The prevalence of infection by at least one type of HPV-AR, except HPV16 and HPV18 (pooled primer with 12 types) was 8.6% (95%CI 7.30–9.77), while the prevalence of HPV infection by HPV 16 and 18 was 1.8% (95%CI 1.20–2.35) and 0.5% (95%CI 0.22–0.86), respectively. The prevalence of coinfection by HPV16 or HPV18 and at least one of the 12 types of pooled primer (31, 33, 35, 39, 45, 51, 52, 56, 58, 59, 66, and 68) was equal to 1.7% (95%CI 1.16–2.29) (Figure).

**Figure:**
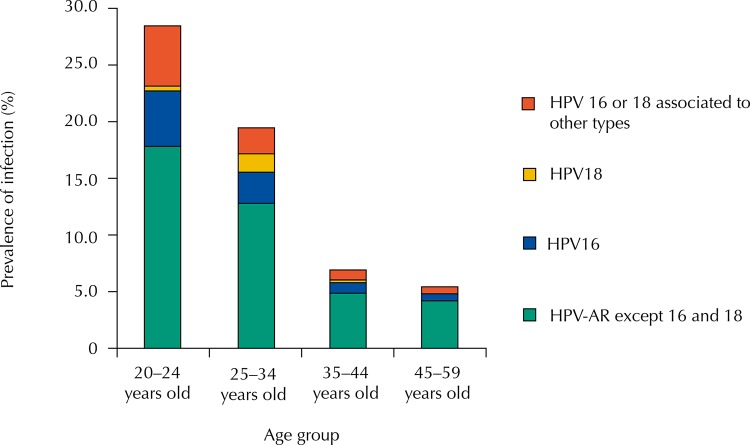
Prevalence of infection by types of HPV according to studied women age groups. Juiz de Fora, State of Minas Gerais.

The prevalence of HPV infection according to selected independent variables is shown in Table 2. In the bivariate analysis, the following variables were associated with higher prevalence of HPV infection: marital status (single: PR = 2.26, 95%CI 1.79–2.84), age range (20–24 years: PR = 5.34, 95%CI 3.65–7.79), education (completed middle school: PR = 2.26, 95%CI 1.79–2.84), health assessment (good to very good: PR = 1.40, 95%CI 1.10–1.79), alcohol consumption (any frequency: PR = 1.89, 95%CI 1.49–2.38) smoker (current: PR = 1.36, 95%CI 1.05–1.76), use of contraceptive (any method: PR = 1.68, 95%CI 1.26–2.23), nulliparity (PR = 2.04, 95% CI 1.57–2.66), age at sexarche ≤ 15 years old (PR = 1.49, 95%CI 1.17–1.92) and sexual partners throughout life (number ≥ 3: PR = 1.84, 95%CI 1.45–2.33).

**Table 2:** Prevalence and gross prevalence ratio of HPV infection according to variables selected among women residing in the coverage area of the Family Health Strategy. Juiz de Fora, State of Minas Gerais, 2010–2012.

Variable	Categories	Total*	HPV+			Gross PR*	95%CI	p

			n	%	95%CI			
Age group (years)	20–24	246	70	28.4	22.90–34.53	5.34	3.65–7.79	< 0.001
	25–34	575	112	19.5	16.31–22.95	3.65	2.54–5.25	< 0.001
	35–44	544	38	7.0	4.99–9.46	1.31	0.84–2.03	0.233
	45–59	657	35	5.3	3.73–7.33	1	-	-
	Total	2,022	255	12.6	11.19–14.13			
Marital status	Had partner	1,601	160	10.0	8.56–11.56	1	-	-
	Single	420	95	22.6	18.70–26.92	2.26	1.79–2.84	< 0.001
Middle school	Incomplete	914	73	8.0	6.31–9.93	1	-	-
	Complete	1,086	179	16.5	14.32–18.82	2.06	1.59–2.67	< 0.001
Skin color	White	951	115	12.1	10.08–14.33	1	-	-
	Non-white	1,071	140	13.17	11.10–15.23	1.08	0.85–1.36	0.508
Low family income	No	1,263	155	12.3	10.51–14.20	1	-	-
	Yes	759	100	13.2	10.84–15.79	1.07	0.84–1.35	0.553
Health assessment	Very poor to regular	842	86	10.2	8.55–12.45	1	-	-
	Good to very good	1,174	169	14.4	12.53–16.43	1.40	1.10–1.79	0.006
Alcohol consumption	No (never)	1,152	105	9.1	7.51–10.92	1	-	-
	Yes	870	150	17.2	14.78–19.91	1.89	1.49–2.38	< 0.001
Current smoker	No	1,569	183	11.7	10.11–13.35	1	-	-
	Yes	434	69	15.9	12.58–19.68	1.36	1.05–1.76	0.018
Former smoker	Yes	465	50	10.7	8.08–13.92	1	-	-
	No	1,160	142	12.2	10.40–14.26	1.13	0.84–1.54	0.403
Menarche ≤ 12 years	Yes	888	107	12.0	9.98–14.37	1	-	-
	No	1,099	142	12.9	10.99–15.04	1.07	0.84–1.35	0.560
Late pap smear	No	1,574	202	12.8	11.21–14.58	1	-	-
	Yes	447	53	11.8	9.00–15.22	0.92	0.69–1.22	0.585
Uses contraceptives	No	638	55	8.6	6.56–11.07	1	-	-
	Yes	1,379	200	14.5	12.68–16.47	1.68	1.26–2.23	< 0.001
Previous pregnancy	Yes	1,772	198	11.2	9.74–12.73	1	-	-
	No	249	57	22.9	17.82–28.61	2.04	1.57–2.66	< 0.001
Sexarche at ≤ 15 years	No	1,559	178	11.4	9.88–13.10	1	-	-
	Yes	438	75	17.1	13.71–20.98	1.49	1.17–1.92	0.001
Up to 3 sexual partners	Yes	1,400	142	10.1	8.61–11.84	1	-	-
	No	530	99	18.7	15.44–22.26	1.84	1.45–2.33	< 0.001
Test - positive for syphilis	No	935	138	14.7	12.54–17.19	1	-	-
	Yes	16	1	6.2	1.58–30.23	0.42	0.63–2.84	0.377
Test - HIV positive	No	175	1,258	13.9	12.04–15.94	1	-	-
	Yes	1	8	12.5	3.15–52.65	0.89	0.14–5.65	0.909

* Total of women with HPV test results and valid information about the variable.

In the multivariate analysis, the following variables remained significantly related to HPV infection: single (PR = 1.40, 95%CI 1.07–1.84), alcohol consumption at any frequency (RR = 1.44, 95%CI, 1.11–1.86) and had three or more sexual partners throughout life (PR = 1.35, 95%CI 1.04–1.74) (Table 3).

**Table 3:** Raw and adjusted HPV infection prevalence ratios for selected variables among women residing in the coverage area of the Family Health Strategy. Juiz de Fora, State of Minas Gerais, 2010–2012.

Variable	Category	Gross PR	95%CI	P	Adjusted PR	95%CI
Age group (years)	45–59	1	-	-	1	-
	35–44	1.31	0.84–2.03	0.233	1.08	0.67–1.74
	25–34	3.65	2.54–5.25	< 0.001	2.66	1.74–4.07
	20–24	5.34	3.65–7.79	< 0.001	3.59	2.23–5.78
Marital status	Married (lifetime)	1	-	-	1	-
	Single (lifetime)	2.26	1.79–2.84	< 0.001	1.40	1.07–1.84
Middle school	Incomplete	1	-	-	1	-
	Complete	2.06	1.59–2.67	< 0.001	1.11	0.82–1.50
Health assessment	Very poor to regular	1	-	-	1	-
	Good to very good	1.40	1.10–1.79	0.006	1.15	0.90–1.48
Alcohol consumption	No (never)	1	-	-	1	-
	Yes	1.89	1.49–2.38	< 0.001	1.44	1.11–1.86
Current smoker	No	1	-	-	1	-
	Yes	1.36	1.05–1.76	0.018	1.23	0.92–1.64
Contraception	No method	1	-	-	1	-
	Any method	1.68	1.26–2.23	< 0.001	1.01	0.75–1.36
Nulliparity	No	1	-	-	1	-
	Yes	2.04	1.57–2.66	< 0.001	1.11	0.80–1.55
Age at sexarche	≥16 years	1	-	-	1	-
	≤ 15 years	1.49	1.17–1.92	0.001	0.95	0.72–1.26
Number of sexual partners	Up to 3 (lifetime)	1	-	-	1	-
	Over 3 (*lifetime*)	1.84	1.45–2.33	< 0.001	1.35	1.04–1.74

## DISCUSSION

The prevalence of cervical HPV infection in women assisted by the Family Health Strategy was 12.6%. This measure is close to the prevalence (12.8%) observed by Girianelli et al. (2010) in a study with the household recruitment of low-income women living in the municipalities of Baixada Fluminense, Rio de Janeiro, which used the hybrid capture technique to detect HPV. Although the PCR technique will present higher prevalence estimates than those obtained using the hybrid capture technique, the measures obtained in the study by Girianelli et al. (2010) resulted from the inclusion of women who had not received preventive examination for more than three years, while the present study considered data from all the women in the target population to be screened^11^ ^,^ ^13^.

The target population women’s attendance to the cervical cancer screening in FHS units participating in the present study was lower than expected (60%). It was expected that, as an action started with household recruitment and appointment scheduling at the health unit, women’s demand would be higher. Reduced adherence to screening reflects the behavior observed in other Brazilian studies and may have influenced the estimates observed for the group. This shows that, even in organized screening, there seem to be factors that influence the response to recruitment, resulting in partial adherence^15^. It should be considered that some women who did not come to the unit may have undergone the preventive examination in services outside the SUS because they have a health plan or even because they paid for the examination in private service. This limitation, however, does not necessarily compromise the representativeness of the findings of the geographical area since there seems to be minor variation in socioeconomic status among resident women.

Regarding losses in HPV testing, despite periodic training and supervision, there were failures in capturing HPV typing and detection and cytology results, with no significant impact on the analysis of results. Such losses were somewhat predicted, considering that the study was operationalized through the health service.

The results concerning the circulating types of HPV in the present study were discordant of some studies that evaluated the prevalence of HPV among Brazilian women^16^. The prevalence of infection by types 16 and 18 is similar to the world prevalence, with HPV16 infection being the most frequent, either isolated or in coinfections, followed by isolated HPV18 infection alone, suggesting a lower prevalence than the types contained in the HPV high-risk pooled primer^4^. It was not possible to identify which types would succeed them virtually in the ecological niche since the pooled primer used included only 12 types of high-risk HPV. However, the prevalence of infection by the types contained in this primer was approximately five times greater than the prevalence of isolated infection by HPV16. Assuming a hypothetical homogeneous distribution between the primer types, this prevalence can be interpreted in a preliminary way as a prevalence of 0.71% for each type, preceding the infection isolated by HPV18 in magnitude and relevance. As this homogeneous distribution certainly does not occur, the difference in prevalence between certain types of high-risk HPV and HPV18 may be even greater.

The prevalence of cervical infection by HPV varies from medium to high, especially among young women, who initiate sexual activity with a risk of exposure to oncogenic types. The incorporation of HPV testing in the screened women would have the advantage of increasing the follow-up interval, with HPV-positive women having a cytological examination, whereas HPV-negative women would only need five-year intervals, two years longer than recommended for follow-up with a cytological examination after two years with normal results^5^ ^,^ ^20^.

The prevalence of HPV infection in Brazilian studies performed with women recruited from health units appears to be considerably higher than the prevalence observed in population-based studies. In the latter, the possibilities of selection bias and consequent overestimation of prevalence estimates are practically eliminated, since the studied population does not only include women who were assisted by, or referenced to, health services for being symptomatic.

Regarding the factors related to cervical HPV infection observed, marital status (single), alcohol consumption (any frequency), and the number of lifetime sexual partners (three or more) remained as independent predictors of HPV infection, even after adjustment for other variables. These findings confirm the results of other studies that also related the risk of HPV infection to marital status, abusive alcohol consumption and the sexual history of the women^1^ ^,^ ^3^ ^,^ ^7^ ^,^ ^8^ ^,^ ^11^ ^,^ ^18^ ^,^ ^23^ ^-^ ^25^ ^,^ ^27^. We did not investigate the association between having extramarital relationships or having partners with extramarital relationships and the prevalence of HPV infection, a factor that has been associated with the prevalence of infection in some studies.

Self-assessment of health status, smoking, contraceptive use, parity and age at sexarche did not remain as independent predictors of HPV infection in the final model. Vaccarella et al. (2006) also found no statistically significant association between HPV infection and parity, prolonged use of oral contraceptives or use of condoms by the sexual partner and age at sexarche^26^ ^,^ ^27^. Smoking has been identified as a factor associated with the persistence and onset of the neoplasia, but not with the risk of infection and its association with HPV infection has been ruled out^28^.

It should be noted that the prevalence of infection in the different age groups is close to that estimated in similar studies, with the highest proportion of infected women found between 25 and 34 years of age, which is also the age group with the highest incidence of in situ carcinoma^17^ ^,^ ^18^ ^,^ ^25^.

Thus, the prevalence of infection among the studied women is similar to that found in other Brazilian studies, being higher among younger women. Independent predictors of HPV infection – marital status, alcohol consumption, and the number of lifetime sexual partners – are highly influenced by economic, cultural, and social issues. Further studies are needed to understand if the relationship between the factors associated with increased prevalence of HPV infection changes when analyzed against the incidence of pre-invasive and invasive cervical cancer lesions among these women to verify whether predictive factors would be the same in a population of women with carcinoma (in situ or invasive).

Organized screening, an alternative to opportunistic screening, provides for the capture of women in the target age groups, while at the same time not unnecessarily repeating the pap smear. However, factors related to non-adherence to the exam within the recommended periodicity need to be recognized and become the target of intervention, since the simple change in the recruitment strategy does not guarantee that the woman will attend the health unit for examination. Testing for HPV, as well as screening, would perform better on an organized, non-opportunistic screening system.

Testing for HPV should be evaluated economically for its implementation to be recommended. As a technology to be incorporated into the screening, it requires team training and strict logistic supervision to avoid contamination of negative samples or cross-contamination by types between samples. It is necessary to adopt methods based on polymerase chain reaction that recognize each type of HPV in the prevalence of infection among the women of the different regions of the country. The higher sensitivity and lower specificity of the HPV-DNA test compared to screening with conventional cytology could overload secondary care referrals. The impact of this increase in positivity on services needs to be evaluated, including its cost-effectiveness ratio. On the other hand, a change in the age range of women screened and the use of the examination in regions of restricted access to health units would benefit women living with unequal access to cervical cancer prevention, identifying women at higher risk for acquisition and persistence of the infection and, consequently, for the evolution of preneoplastic lesions. The ethical implications of HPV testing need to be considered, and the psychological impact judged carefully, since women who have HPV infection are not ill, and it may be hard to understand this condition. Screening provides an overview of the HPV infection prevalence at a time when circulatory type surveillance is needed, including ecological niche filling, subsequent to vaccine implantation against types 16 and 18, and this action can be viewed as an effort within priorities of policies aimed at the control of cervical cancer and, therefore, at women’s health.

In general, actions that focus on the social determinants of health, course approach, quality of life, and lifestyle promote improvements that are essential to modifying exposure to risk factors related to HPV infection, as well as to many other exposures, diseases, and aggravations. Gender-related issues and their vulnerabilities should guide the planning of educational actions among women, permeating prevention and control of injuries even before sexual activity begins. The health sector must work in an integrated manner with other sectors to ensure that inequalities in access to skilled services and information on women’s health are overcome and to identify opportunities for integration. The search for a solution to these problems will not be complete without dialogue between epidemiological research and health service, in a mutually beneficial process, where the most favored will certainly be the female population.
